# Multimodality Imaging Evaluation of an Uncommon Entity: Esophageal Heterotopic Pancreas

**DOI:** 10.1155/2014/614347

**Published:** 2014-03-11

**Authors:** Takman Mack, Debra Lowry, Peter Carbone, Brian Barbick, Joshua Kindelan, Robert Marks

**Affiliations:** ^1^Department of Radiology and Nuclear Medicine, Naval Medical Center, 34800 Bob Wilson Drive, San Diego, CA 92134, USA; ^2^Department of General Surgery, Naval Medical Center, 34800 Bob Wilson Drive, San Diego, CA 92134, USA; ^3^Department of Pathology, Naval Medical Center, 34800 Bob Wilson Drive, San Diego, CA 92134, USA; ^4^Department of Cardiothoracic Surgery, Naval Medical Center, 34800 Bob Wilson Drive, San Diego, CA 92134, USA

## Abstract

A 25-year-old male was referred to the Radiology Department with new onset of right upper quadrant and epigastric abdominal pain. He had no past medical or surgical history. Physical exam was unremarkable. The patient underwent computed tomography (CT), fluoroscopic upper gastrointestinal (GI) evaluation, endoscopic ultrasound (EUS), and positron emission tomography (PET) evaluation, revealing the presence of a heterogeneous esophageal mass. In light of imaging findings and clinical workup, the patient was ultimately referred for thorascopic surgery. Surgical findings and histology confirmed the diagnosis of esophageal heterotopic pancreas.

## 1. Introduction 

Heterotopic pancreas is defined as histologically proven normal pancreatic tissue that lacks anatomic, vascular, and ductal continuity with the orthotopic pancreas. Other common names for this entity include ectopic, aberrant, or accessory pancreas [1]. Heterotopic pancreas is most commonly discovered incidentally in the abdomen. However, intrathoracic location in association with the esophagus is rare, with only 15 cases reported in the English literature [2]. Here, we report a very rare case of esophageal heterotopic pancreas discovered after extensive radiologic investigation and pathologic correlation.

## 2. Case Presentation 

A 25-year-old male with no significant past medical or surgical history presented with 2 weeks of moderate to severe right upper quadrant and epigastric abdominal pain. His pain was occasionally associated with heavy exercise and meals. Physical exam and clinical laboratory findings were unremarkable.

**Figure 1:**
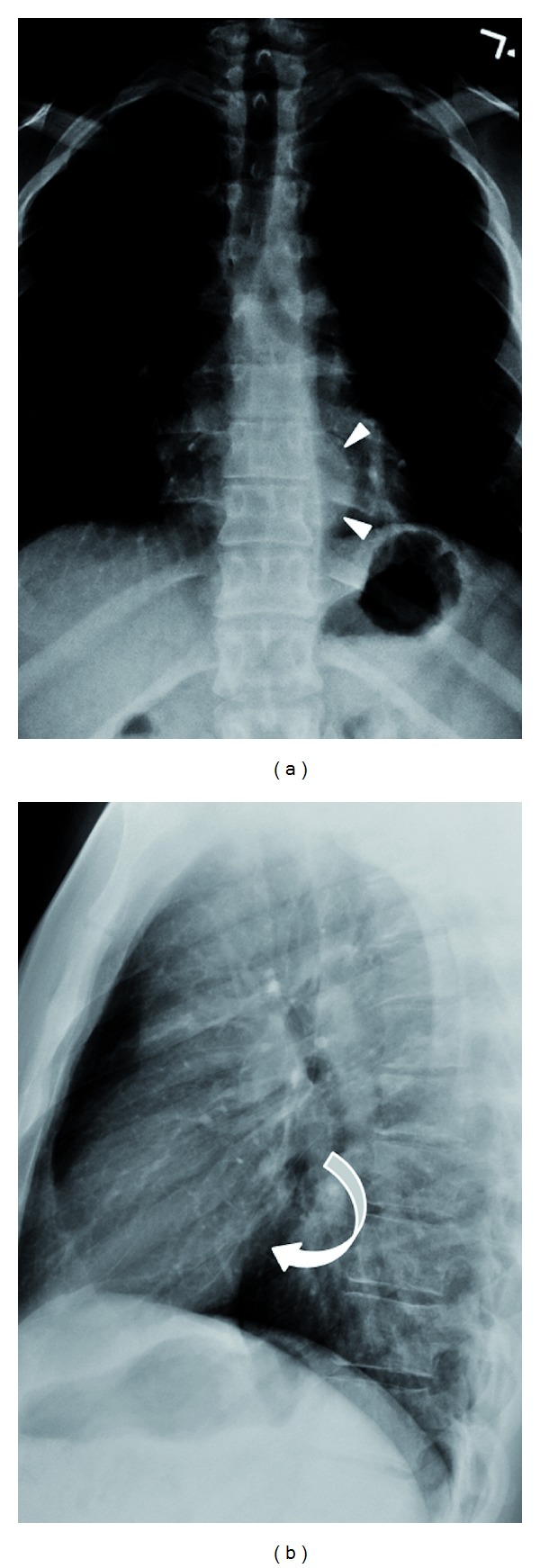
(a) Frontal radiograph shows the presence of a hemispheric, retrocardiac opacity superior to the left cardiophrenic sulcus (arrowheads). (b) Lateral radiograph demonstrates a subtle rounded lucency marginating the posterior aspect of the heart (curved arrow).

Initial radiographic evaluation demonstrated abnormal frontal and lateral chest images (Figures 1(a) and 1(b)). Following the descending aortic shadow inferiorly, a subtle opacity with a rounded contour was identified superior to the gastroesophageal (GE) junction. This was not well appreciated on the lateral view, possibly due to the overlapping shadow of the posterior mediastinum. These radiographic findings were suspicious for a retrocardiac posterior mediastinal mass.

**Figure 2:**
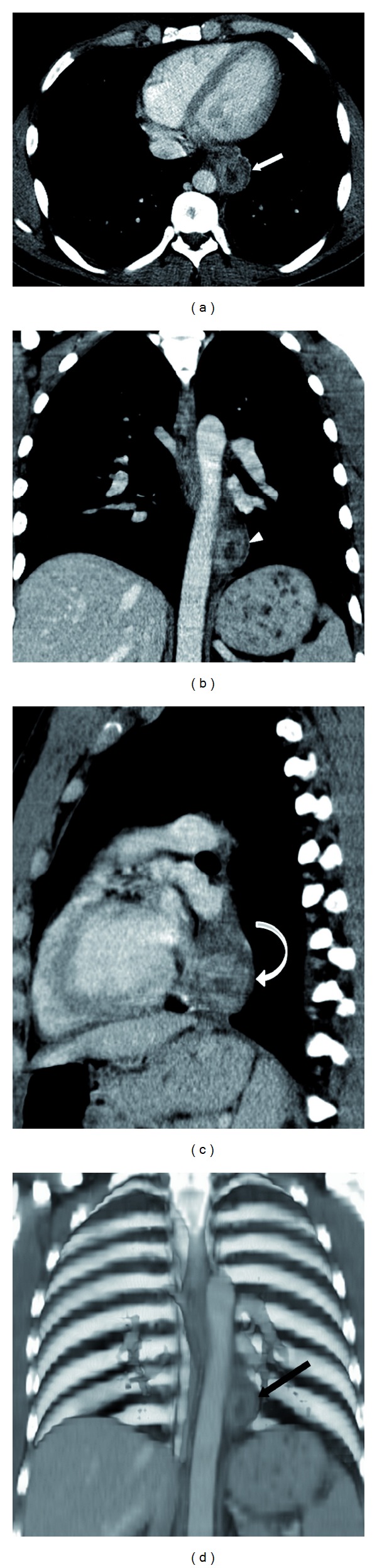
(a) Contrast enhanced CT axial image showing the presence of a rounded mass with central hypoattenuation adjacent to the esophagus (arrow). Images obtained on a Siemens Somatom Sensation 64 slice after administration of intravenous contrast (Isovue 370) 100 mL. Total DLP 1128.81 mGycm. (b) Coronal multiplanar reformatted CT image showing the mass adjacent to the thoracic aorta (arrowhead). (c) Sagittal multiplanar reformatted CT image showing the retrocardiac mass (curved arrow). (d) Volume rendered coronal reformatted CT image showing the central hypoattenuation within the paraesophageal mass (black arrow).

**Figure 3:**
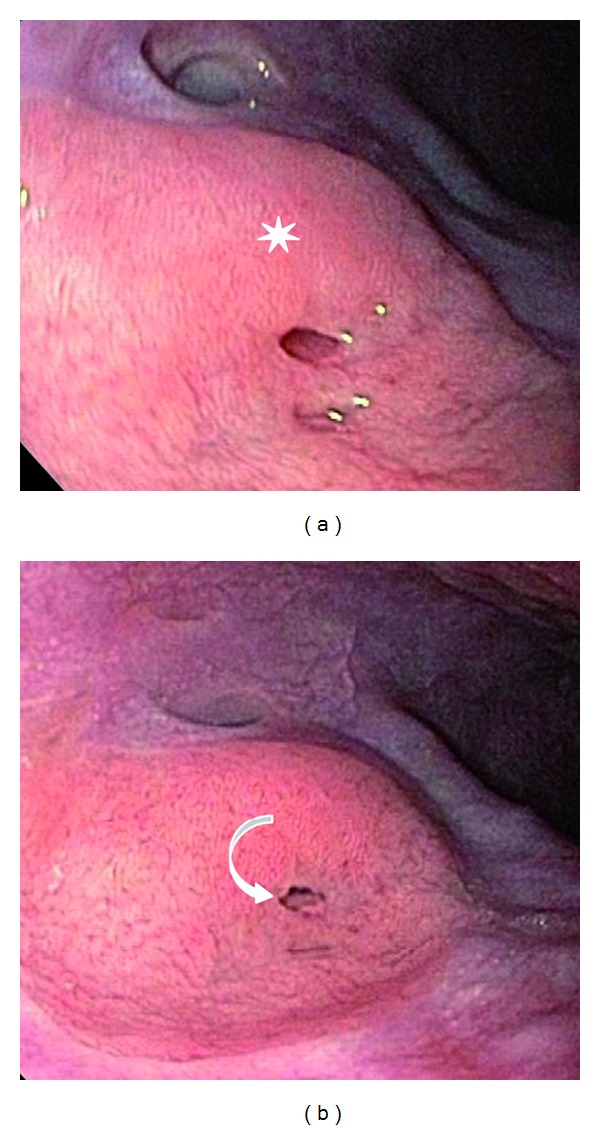
(a) Still photograph obtained during endoscopy in the region superior to the gastroesophageal junction showing the presence of a subepithelial mass with numerous holes in the overlying epithelium (white star). (b) Another endoscopic still image of the subepithelial mass with possible rudimentary ductal structure (curved arrow).

**Figure 4:**
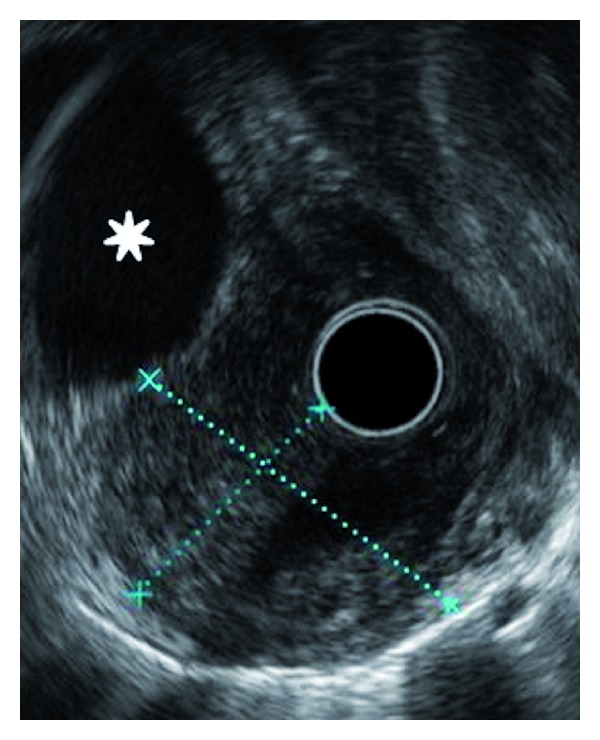
Endoscopic sonographic image showing well-circumscribed hypoechoic structure in the distal esophagus (blue calipers). There is broad abutment with the aorta (small star).

Given the vague right upper quadrant and epigastric symptoms, a right upper quadrant ultrasound (US) was performed. However, the exam was noncontributory. Continued suspicion for a mass prompted further evaluation with computed tomography (CT) (Figures 2(a), 2(b), 2(c), and 2(d)). A 4.4 × 3.7 × 3.2 cm paraesophageal mass was demonstrated just superior to the gastroesophageal (GE) junction. The mass consisted of a peripheral rim of heterogeneous tissue (1 cm in thickness) with moderate enhancement and a central ovoid region of lower density (average 19 Hounsfield units). No local or distant lymphadenopathy was present. No discrete fistulous esophageal connection was identified; however, the mass demonstrated broad abutment along the distal esophagus with evidence of mild contouring and adjacent luminal irregularity and narrowing. The imaging characteristics (size, attenuation, contours, and enhancement) were most consistent with a subepithelial mass, though the tissue of origin remained uncertain. Given the patient demographics, tumor size, and CT appearance, a gastrointestinal stromal tumor was initially favored. Due to the distal esophageal location, heterotopic pancreas was not initially considered in the differential diagnosis. Subsequently, esophagogastroduodenoscopy (EGD) visualized multiple small holes in the esophageal wall overlying the mass, thought to represent fistulous tracts (Figures 3(a) and 3(b)). Biopsies in this region were performed; however, they were ultimately deemed nondiagnostic. Endoscopic ultrasound (EUS) revealed a well-circumscribed heterogeneous hypoechoic subepithelial mass abutting the aorta (Figure 4(a)). Though the EGD and EUS findings were nonspecific, the apparent fistulous tracts raised the suspicion of abnormal connection with an airway or possibly with another subjacent anatomic structure.

**Figure 5:**
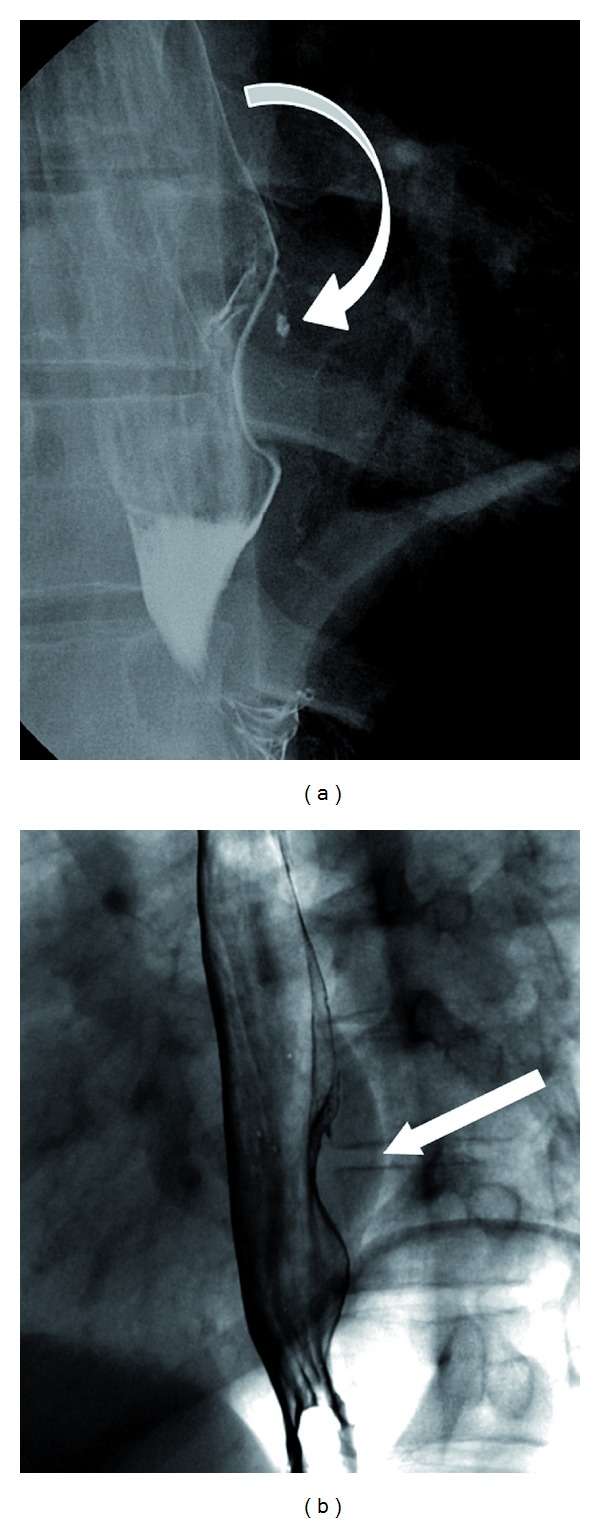
(a) Double-contrast UGI fluoroscopic oblique image showing the presence of a subepithelial mass with small extraluminal oral contrast (curved arrow). Images obtained on a Siemens AXIOM Luminos TF, total fluoroscopic time for the exam: 4.1 minutes. (b) UGI oblique image demonstrating a smooth-surfaced, hemispheric lesion forming obtuse angles with the adjacent luminal contour (arrow).

An upper gastrointestinal (UGI) double contrast fluoroscopic exam was performed to further characterize the subepithelial mass and to investigate the apparent fistulas. Upright frontal and left anterior oblique low-dose fluoroscopic images demonstrated classic UGI findings of a subepithelial mass: a smooth-surfaced, hemispheric lesion forming obtuse angles with the adjacent luminal contour [3] (Figures 5(a) and 5(b)). Interestingly, the UGI series also demonstrated trace slivers of extraluminal contrast, presumably representing extension into the tumor proper. Notably, there was no evidence of contrast extravasation within the airways, subpleural space, or lung parenchyma.

**Figure 6:**
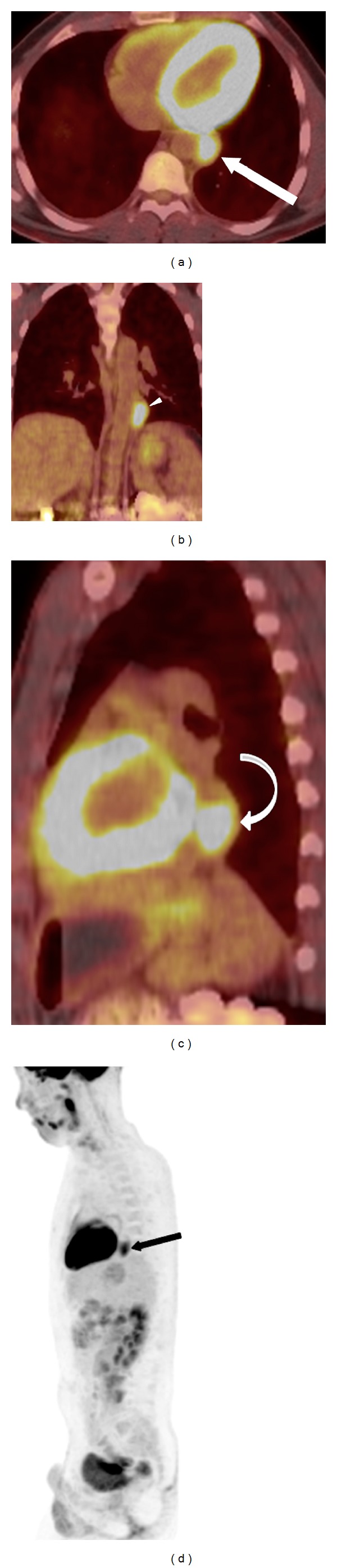
(a) PET utilizing fluorine-18 fluorodeoxyglucose (F-18 FDG) axial fused CT image showing the increased FDG avidity associated with the paraesophageal mass (arrow). Images obtained on a Siemens Biograph PET CT 64 slice after administration of 12.31 mCi F-18 FDG. Total DLP 447 mGycm. (b) Coronal multiplanar reformatted fused PET CT image showing the FDG avid mass adjacent to the thoracic aorta (arrowhead). (c) Sagittal multiplanar reformatted fused PET CT image showing the FDG avid retrocardiac mass (curved arrow). (d) Maximal intensity projection (MIP) fused PET CT image showing the retrocardiac mass (black arrow).

Despite extensive imaging and numerous biopsy attempts, the differential diagnosis remained broad and included both benign and malignant processes. Considering the possibility of malignancy, a positron emission tomography (PET) scan was performed utilizing fluorine-18 fluorodeoxyglucose (F-18 FDG). The mass demonstrated increased FDG avidity, with a standardized uptake value average of 6.0 and maximally of 10.0 (Figures 6(a), 6(b), 6(c), and 6(d)). The remainder of the study demonstrated physiologic uptake. The associated FDG uptake immediately raised concern for a neoplasm. However, FDG is not a cancer-specific agent and uptake can be seen with inflammatory, infectious, and neoplastic processes. Moreover, normal uptake of FDG commonly occurs in various sites throughout the body. The gastroesophageal junction region is known to occasionally show normal uptake [4].

**Figure 7:**
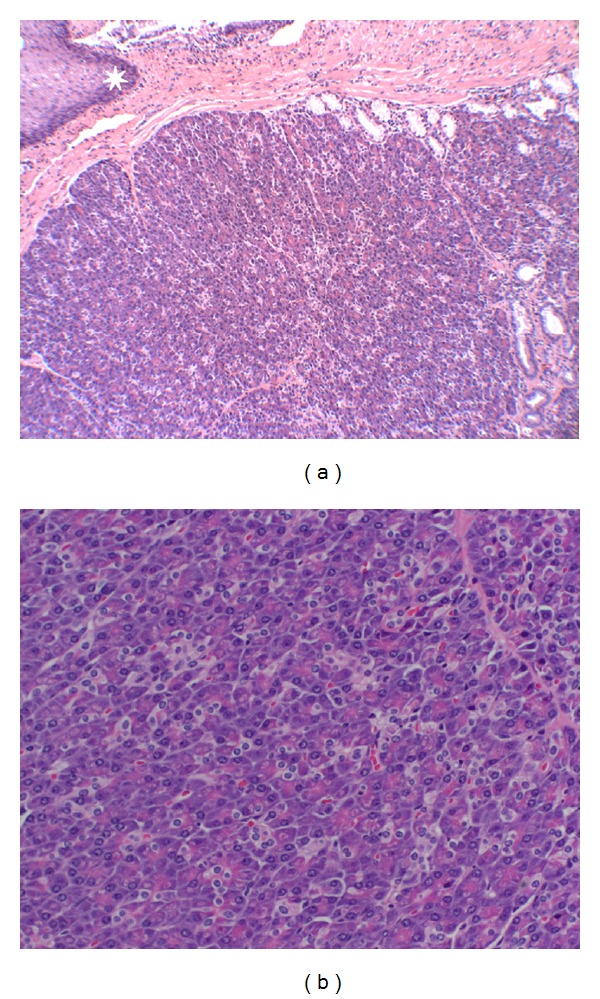
(a) Hematoxylin and eosin (H&E) stain of the esophageal excisional biopsy demonstrating a lobule of heterotopic pancreatic acini and adjacent fibrous septa containing intralobular pancreatic ducts (lower right portion of image). Overlying esophageal squamoglandular mucosa is present (upper left white star). Magnification: 10x objective. (b) Heterotopic pancreatic acini. Several pyramid-shaped cells arranged concentrically around a central duct form an acinus.

In the absence of a tissue diagnosis or diagnostic imaging, optimal management remained unclear. In order to obtain definitive tissue diagnosis, left-sided video-assisted thoracic surgery was performed which revealed a soft, mobile, cyst-like structure upon direct visualization. Pathologic analysis demonstrated presence of pancreatic heterotopia with overlying unremarkable squamoglandular mucosa (Figures 7(a) and 7(b)). The pathologic findings excluded the possibility of metaplasia, dysplasia, or malignancy.

## 3. Management 

Management of this entity has varied from observation to radical surgery, such as Ivor-Lewis esophagectomy. As evidenced by our case, preoperative diagnosis is often difficult and is usually determined once the specimen is removed in its entirety. After weighing the severity of symptoms and the possibility for malignant potential, surgical resection of heterotopic pancreas is often the preferred management [5]. While the lesion appeared relatively benign under direct visualization, complete surgical resection was considered appropriate in our case.

## 4. Follow-Up 

The patient had an uneventful surgical recovery. A swallow study performed on postoperative day three showed no evidence of contrast extravasation (images not shown). He had an uneventful hospital course, and the patient returned for two-month clinic follow-up without surgical complications and self-reported resolution of the epigastric pain.

## 5. Discussion 

Heterotopic pancreas, in any location, is found in 0.6% to 13.6% of autopsies and 0.2% to 0.5% of abdominal operations [6]. There is a slight predilection to occur in males, generally in the fourth to the sixth decades of life. Esophageal heterotopic pancreas is a rare entity, with a comprehensive literature review revealing only fifteen cases reported to date.

Our case demonstrated a classic presentation of subepithelial mass on UGI fluoroscopic evaluation. Subepithelial masses, previously termed “submucosal” masses, are defined as masses covered with normal appearing submucosa. “Subepithelial” is the preferred term because these masses can arise from any layer of the gastrointestinal (GI) wall (i.e., intramural). In addition, other intra-abdominal structures and masses (i.e., extramural) can produce extrinsic impressions visible on imaging [7]. The PET CT findings were somewhat confounding, but the noted FDG uptake likely represented a localized inflammatory process, consistent with the patient's symptomatology.

Although typically asymptomatic, esophageal heterotopic pancreas has been associated with epigastric pain, dysphagia, obstruction, and upper gastrointestinal bleeding [8]. As a normal eutopic pancreatic tissue, heterotopic pancreatic tissue specimens can contain pancreatic acini, ducts, and occasionally islets of Langerhans [9]. Pancreatic acini make up the majority of normal pancreatic tissue and are responsible for the exocrine function of the organ. Acinar cells contain membrane-bound zymogen granules, rich in digestive enzymes [10]. This enzyme-rich serous fluid is then secreted into the duct lumen. Acinar cells are normally pyramidally shaped epithelial cells that are radially oriented around a central lumen. In retrospect, the central hypodensity seen on CT could have represented a rudimentary ductal structure.

The normal pancreas arises from fusion of the ventral and dorsal outpouchings of the foregut [10]. Although the pathogenesis of heterotopic pancreas has not been definitively established, a current theory postulates that the embryologic origin involves abnormal detachment of one or more branching pancreatic buds during embryonic rotation. Following this theory, it is not surprising that heterotopic pancreas most commonly occurs near the stomach; specifically, in the prepyloric region along the greater curvature [1]. Complications of heterotopic pancreas include pseudocyst formation, pancreatitis, and both benign and malignant transformation [11]. A small case series reported malignancy in two out of nine observed cases of esophageal heterotopic pancreas [6]. Potential neoplasms associated with ectopic pancreas include adenocarcinomas, anaplastic cancers, islet cell adenomas, cystadenocarcinomas, and solid and papillary epithelial tumors [6]. Though exceedingly rare, our case of esophageal heterotopic pancreas displayed an excellent review of several classic radiographic findings while emphasizing the importance of maintaining a broad differential diagnosis.

## Abbreviations

UGI: Upper gastrointestinal exam
GI: Gastrointestinal
EGD: Esophagogastroduodenoscopy
PET: Positron emission tomography
CT: Computed tomography
US: Ultrasound
GE: Gastroesophageal
SUV: Standardized uptake value
F-18 FDG: Fluorine-18 fluorodeoxyglucose
MIP: Maximal intensity projection.
